# Detecting alpha spindle events in EEG time series using adaptive autoregressive models

**DOI:** 10.1186/1471-2202-14-101

**Published:** 2013-09-18

**Authors:** Vernon Lawhern, Scott Kerick, Kay A Robbins

**Affiliations:** 1 Department of Computer Science, University of Texas at San Antonio, San Antonio, TX 78249, USA; 2 Human Research and Engineering Directorate, US Army Research Laboratory, Aberdeen Proving Ground, Aberdeen, MD 21005, USA

**Keywords:** Alpha spindle, Adaptive autoregressive model, Classification, Electroencephalography, Time series, Fatigue, Change point detection

## Abstract

**Background:**

Rhythmic oscillatory activity is widely observed during a variety of subject behaviors and is believed to play a central role in information processing and control. A classic example of rhythmic activity is *alpha spindles*, which consist of short (0.5-2 s) bursts of high frequency alpha activity. Recent research has shown that alpha spindles in the parietal/occipital area are statistically related to fatigue and drowsiness. These spindles constitute sharp changes in the underlying statistical properties of the signal. Our hypothesis is that change point detection models can be used to identify the onset and duration of spindles in EEG. In this work we develop an algorithm that accurately identifies sudden bursts of narrowband oscillatory activity in EEG using techniques derived from change point analysis. Our motivating example is detection of alpha spindles in the parietal/occipital areas of the brain. Our goal is to develop an algorithm that can be applied to any type of rhythmic oscillatory activity of interest for accurate online detection.

**Methods:**

In this work we propose modeling the alpha band EEG time series using discounted autoregressive (DAR) modeling. The DAR model uses a discounting rate to weigh points measured further in the past less heavily than points more recently observed. This model is used together with predictive loss scoring to identify periods of EEG data that are statistically significant.

**Results:**

Our algorithm accurately captures changes in the statistical properties of the alpha frequency band. These statistical changes are highly correlated with alpha spindle occurrences and form a reliable measure for detecting alpha spindles in EEG. We achieve approximately 95% accuracy in detecting alpha spindles, with timing precision to within approximately 150 ms, for two datasets from an experiment of prolonged simulated driving, as well as in simulated EEG. Sensitivity and specificity values are above 0.9, and in many cases are above 0.95, for our analysis.

**Conclusion:**

Modeling the alpha band EEG using discounted AR models provides an efficient method for detecting oscillatory alpha activity in EEG. The method is based on statistical principles and can generally be applied to detect rhythmic activity in any frequency band or brain region.

## Background

Alpha waves ([8, 13] Hz) were among the earliest described functional oscillatory components in the human EEG [1], and research has supported the general notion that alpha band power is inversely related to brain activation [2-4] and reflects deactivated or inhibited cortical processes [5]. Several different alpha rhythms have been identified (e.g., mu, sigma, tau, occipital) across various brain regions. In particular, rhythmic alpha activity has been observed to increase in posterior brain regions (parietal-occipital) during attentional lapses [6,7] and during states of drowsiness relative to states of alertness [8-11].

A widely-studied characteristic of the alpha frequency band is the *alpha spindle,* a large narrowband burst of alpha activity that usually occurs over short (0.5-2 s) duration [8,11,12]. Alpha spindle spectral microstructures have peak amplitudes generally in the higher alpha frequencies ([10, 13] Hz) and have characteristics that resemble the “waxing and waning” of the alpha rhythm [12,13]. Alpha spindles also have been shown to occur primarily in the parietal/occipital regions of the brain and have been correlated with fatigue, drowsiness and reduced driving performance in experiments of prolonged driving [11,12]. Several methods have been developed to characterize alpha power changes associated with various tasks. These methods include fast Fourier transforms (FFT) [7,10,11], wavelets [14], phase [15], matching pursuit [16,17], ERD/ERS [5], autoregressive modeling [6,18,19], adaptive filtering [20], neural network analysis [21], fuzzy systems [22,23], and nonlinear EEG analyses [7,24]. Other characterizations of alpha activity are the alpha band power of the signal [10] and power ratios such as the (alpha + theta)/beta ratio.

Recently, Simon et al. [11] developed an algorithm specifically for detecting alpha spindles in EEG by calculating the full width at half maximum (FWHM) of the amplitude spectral density of the alpha frequency range. Features of alpha spindles, such as alpha spindle rate and alpha spindle duration, were extracted from the EEG and correlated with fatigue and drowsiness in both real [11] and simulated [12] driving tasks. However, Simon et al. only validated algorithm accuracy in capturing high frequency narrowband alpha on simulated data and not real EEG. Techniques based on matching pursuit (MP) have also been used by Schönwald et al. to identify alpha spindles in sleep by using a dictionary of Gabor, Fourier and Dirac delta functions [25]. They report sensitivity and specificity values of approximately 0.812 in detecting alpha sleep spindles across all stages of sleep. By changing some of the parameters in their model, they were able to increase the sensitivity and specificity to about 0.9; however, this change increased computational time exponentially.

The goal of this work is to develop an efficient algorithm to reliably detect sudden increases of narrowband oscillatory EEG activity with good temporal resolution. Our motivating example for the development of this algorithm is detecting alpha spindles in EEG. We hypothesize that alpha spindles in EEG represent a changes in the underlying neural dynamics that can be characterized and identified by their statistical properties. To detect these changes, we propose a method that is based on *change-point detection*, a class of time series models designed to discover changes in the underlying statistical properties of time series data [26-30]. These models aim to detect statistical irregularities in data (called change points) with high temporal resolution. Change point detection models have been used previously in many applications such as detecting denial of service (DoS) attacks by monitoring packet activity in computer networks [27,31] and monitoring stock prices [29].

Since EEG signals are highly dynamic, we develop a change-point detection algorithm based on *discounted autoregressive (DAR) models* to represent the EEG time series adaptively in time. DAR models weigh points observed further in the past less heavily than points more recently observed, so they can adapt to the non-stationary nature of EEG. Features that are extracted from a DAR model are time dependent and can be used for analysis of transient EEG. The DAR model can also be updated sequentially in time using an algorithm called the *sequential discounted autoregressive (SDAR) algorithm*[27,28]. This allows for the analysis and monitoring of EEG signals in near real-time, making it a computationally efficient method for EEG analysis.

We apply our SDAR algorithm together with predictive loss scoring to identify time periods in the alpha frequency range of EEG where the time-dependent DAR model cannot adequately describe future data points (periods of high loss scores) and correlate these time periods with alpha spindles in EEG. The model parameters, such as the time-varying AR model coefficients and the model variance, can be used for further analysis of these time periods. We demonstrate the efficacy of this approach both for simulated data as well as for expert-labeled EEG data from simulated driving tasks.

## Methods

### Sequential discounted AR algorithm (SDAR)

Autoregressive models (AR) represent each data point as a linear combination of a certain number (the model order) of previous data points. Discounted AR models assume that data points observed further in the past contribute less information than points more recently observed. An algorithm for the sequential computation of the DAR model parameters was proposed in [28]. We adapt this algorithm for online detection of rhythmic oscillatory activity in EEG, with applications to detecting alpha spindles.

First, we give a description of the standard autoregressive model. Suppose *x*_*t*_ is a zero-mean time series vector of length *n*. AR(*p*), the autoregressive model of order *p*, can be written as:

$$x_{t}=\overset{p}{\underset{i=1}{\sum }}A_{i}x_{t-i}+\varepsilon_{t}$$

where the *A*_*i*_, *i = 1, …, p* denote the AR model coefficients, and *ε*_*t*_ is normally distributed noise with mean 0 and variance *σ*^2^, i.e., *N*(0,*σ*^2^). The temporal dynamics of the time series are described by the model coefficients  *θ* = (*A*_1_, …, *A*_*p*_, *σ*^2^). The model structure given above implies that *P*(*x*_*t*_|*x*_*t* − 1_,.., *x*_*t* − *p*_, *θ*) ~ *N*(*w*, *σ*^2^):

$$P\left(x_{t}|x_{t-1},\ldots ,x_{t-p},\theta \right)=\frac{1}{\sqrt{2\pi \sigma^{2}}}e^{-{\left(x_{t}-w\right)}^{2}/2\sigma^{2}}$$

where $w=\overset{p}{\underset{i=1}{\sum }}A_{i}x_{t-i}$. While there are a variety of methods developed to estimate AR model coefficients, including the Yule-Walker method and the Burg method [32], a straightforward approach is to use Maximum Likelihood Estimation (MLE). MLE estimates the parameters of the model by maximizing the log-likelihood:

$$\hat{\theta }={\mathrm{argmax}}_{\theta }\log L\left(\theta |x_{1},\ldots ,x_{n}\right).$$

The log-likelihood function for the AR(*p*) model can be reduced to:

$$\begin{array}{l} {\;\mathrm{argmax}}_{\theta }\log\left(L\right)={\mathrm{argmax}}_{\theta }\log\left(\overset{n}{\underset{i=p+1}{\Pi }}P\left(x_{i}|x_{i-1},\ldots ,x_{i-p},\theta \right)\right)\;\\ \;={\mathrm{argmax}}_{\theta \;}\overset{n}{\underset{i=p+1}{\sum }}\log P\left(x_{i}|x_{i-1},\ldots ,x_{i-p},\theta \right) \end{array}$$

This procedure is equivalent to minimizing the sum of squared errors for linear Gaussian models:

$$\hat{A}{=\mathrm{argmin}}_{A}\overset{n}{\underset{i=p+1}{\sum }}{\left(x_{i}-A^{T}\;{\bar{x}}_{i}\right)}^{2}\;$$

where $\hat{A}$ is the estimate of the AR model parameters A = (*A*_1_,…,*A*_*p*_)^T^ and ${\bar{x}}_{t}={\left(x_{t-1},\ldots ,x_{t-p}\right)}^{T}$. The variance *σ*^2^ can be estimated in a similar fashion [33].

Discounted autoregressive models instead minimize:

$${\hat{A}}_{t}={\mathrm{argmin}}_{A}\overset{t}{\underset{i=p+1}{\sum }}{\left(1-r\right)}^{t-i}{\left(x_{i}-A_{t}^{T}\;{\bar{x}}_{i}\right)}^{2}$$

where *r* ε(0,1) is the *discounting rate*, and ${\hat{A}}_{t}$ is the discounted maximum likelihood estimate of the DAR model parameters. This model implies that time points observed earlier in the sequence have less influence on the overall likelihood than points observed more recently by a factor of (1-*r*)^*t-i*^. This model has been used previously in the analysis of time series signals in information network security [27,28].

Using the DAR model, Urabe et al. [28] have derived an algorithm for the sequential optimization of the DAR model parameters, called the *sequential discounted autoregressive* (SDAR) *algorithm*. The SDAR algorithm sequentially estimates the DAR model parameters $\left({\hat{A}}_{t},{\hat{\sigma }}_{t}^{2}\right)$ given a new data point in the time series at time *t*. The DAR model parameters have a subscript *t* to emphasize that they are now time-dependent. We use this algorithm to obtain the coefficients of the DAR model, which are used to calculate the mean and variance of the Gaussian distribution that describes the data at each time point. We then calculate a predictive loss function that measures how well the data is modeled by past data points.

Here we define the SDAR algorithm (using the notation from [28,34]). Let *x*_*t*_ be the data point observed at time *t* and let ${\bar{x}}_{t}={\left(x_{t-1},\ldots ,x_{t-p}\right)}^{T}$. Set the DAR model order *p* and the discounting rate *r*. The goal is to estimate the DAR model parameters $\left(A_{t},\sigma_{t}^{2},\mu_{t}\right)$ at each time *t.* The algorithm is given in 5 steps:

1. Initialization of parameters at *t* = *p*:

$$\begin{array}{l} \begin{array}{cc} V_{t}=I_{p} & \;\left(p\times p\right) \end{array}\\ \begin{array}{cc} c_{t}=r{\bar{x}}_{t}^{T}{\bar{x}}_{t} & \;\left(1\times 1\right) \end{array}\\ \begin{array}{cc} M_{t}={\hat{A}}_{\mathrm{burg}} & \left(p\times 1\right) \end{array}\\ \begin{array}{cc} \sigma_{t}^{2}={\hat{\sigma }}_{\mathrm{burg}}^{2} & \;\left(1\times 1\right) \end{array}\\ \begin{array}{cc} A_{t}=V_{t}M_{t} & \;\left(p\times 1\right) \end{array} \end{array}$$

2. Update the model parameters at time *t* = *p* + 1 by calculating:

$$\begin{array}{l} c_{t}=r\;{\bar{x}}_{t}^{T}V_{t-1}\;{\bar{x}}_{t}\\ M_{t}=\left(1-r\right)M_{t-1}+r\;{\bar{x}}_{t}x_{t}\\ V_{t}=\left(\frac{1}{1-r}\right)V_{t-1}-\left(\frac{r}{1-r}\right)\frac{\left(V_{t-1}\left({\bar{x}}_{t}\;{\bar{x}}_{t}^{T}\right)V_{t-1}\right)}{1-r+c_{t}}\;\\ A_{t}=V_{t}M_{t} \end{array}$$

3. Update the mean *μ*_*t*_ and variance $\sigma_{t}^{2}$ of the model:

$$\begin{array}{l} \mu_{t}=A_{t}^{T}\;{\bar{x}}_{t}\\ \sigma_{t}^{2}=\left(1-r\right)\;\sigma_{t-1}^{2}+r{\left(x_{t}-\mu_{t}\right)}^{2} \end{array}$$

4. Calculate the quadratic loss score *ψ*_*t*_ by comparing the current data point to the mean of the Gaussian distribution updated in Step 3:

$$\psi_{t}={\left(x_{t}-\mu_{t}\right)}^{2}$$

5. Repeat Steps 2–4 until the end of the time series at *t* = *n*.

In Step 1 we initialize the parameters at time *t* = *p*, where *p* is the model order. Here, *I*_*p*_ denotes a *p* × *p* identity matrix. We initialize the parameters *M*_*t*_ and $\sigma_{t}^{2}$ by the Burg estimates of the AR parameters and the noise variance, ${\hat{A}}_{\mathrm{burg}}$ and ${\hat{\sigma }}_{\mathrm{burg}}^{2}$, respectively, from an available training dataset, as these values produce stable algorithm performance. Usually we use an initial portion of the time series as training and estimate these values using a normal AR model.

In Step 2 we update the DAR parameters *A*_*t*_ given the new data point *x*_*t*_. Step 3 updates the mean and variance of the Gaussian distribution using the newly estimated DAR model parameters. Step 4 calculates a quadratic loss score by comparing the data point to the mean of the Gaussian distribution at the current time. Steps 2–4 are repeated until the end of the recording at *t* = *n*. We then apply a temporal smoothing function to the quadratic loss score time series to reduce the impact of isolated outliers [27]. We smooth the loss score time series using the mean of five (5) data points.

Alpha spindles in EEG are identified by thresholding the smoothed loss score time series. We find the optimal threshold value by maximizing a weighted F-measure given as:

$$F_{\beta }=\left(1+\beta^{2}\right)\frac{\mathrm{precision}\times \mathrm{recall}}{\left(\beta^{2}\mathrm{precision}+\mathrm{recall}\right)}\;$$

where *β* is a non-negative number, and the precision and recall are given as:

$$\begin{array}{l} \mathrm{precision}=\frac{\mathrm{TP}}{\mathrm{TP}+\mathrm{FP}}\\ \;\mathrm{recall}=\frac{\mathrm{TP}}{\mathrm{TP}+\mathrm{FN}} \end{array}$$

We calculate the True Positive (TP), False Positive (FP), and False Negative (FN) rates by comparing the alpha spindle time regions identified by an expert with those marked by the SDAR algorithm as having a smoothed loss score that exceeds the specified threshold. We use the weighted F-measure to take into account the highly unbalanced nature of the data, as alpha spindles occurred less than 1% of the total time. *β* = 1 reduces to the standard F-measure, which weights precision and recall equally. *β* = 0.5 emphasizes precision more than recall, and *β* = 2 emphasizes recall more than precision. We use *β* = 2 for this analysis to identify the loss score threshold value that produces a minimal number of false negatives. We split our data into two continuous equal halves; one half was used for finding the optimal threshold parameters, while the second half was used for validation. Note that the number of alpha spindles in each half may not be the same.

**Figure 1:**
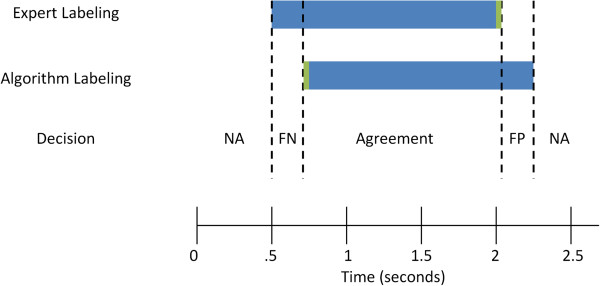
**An illustration of the comparison algorithm for comparing two labeled data sets.** There are 4 possible decisions: NA (Null Agreement), FN (False Negative), FP (False Positive) and Agreement. An optional parameter, the fuzzy window time (shown in green), can be used to account for small timing errors in the comparison.

To compute a direct time comparison between expert labeling and regions marked by the SDAR algorithm as exceeding the threshold, we use the compareLabels function from the DETECT Toolbox [35] for MATLAB (The Mathworks, Natick, MA). An illustration of the comparison algorithm is swn in Figure 1. There are four possible decision types: Null Agreement or NA (agreement of no event present), False Negative or FN (the algorithm fails to identify the expert-labeled time region), False Positive or FP (the algorithm identifies a time region that the expert does not), and Agreement (both the expert and algorithm agree on the time interval). A summary of the comparison is reported by summing the total times in each category. From these values the precision, recall, sensitivity, specificity, and the receiver operating characteristic (ROC) curve can be calculated. The compareLabels function has an optional fuzzy window parameter that can control for minor timing differences between the algorithm labeling and an expert labeling (see [35] for more information).

We report the hit rate (HR) of the algorithm, which is the number of times the algorithm identified a region that was also identified by the expert, regardless of the timing precision in the detected regions. We also report the Spindle Temporal Error (STE) as the ratio of the total time in the False Negative state to the total number of alpha spindles. This gives a summary of the temporal localization of the detected spindles.

If more than one EEG channel is modeled, the SDAR algorithm uses a voting strategy that only selects a time region if a certain percentage of the overall number of channels identified the same time region. This strategy reduces the impact of isolated outliers that may exist only in one EEG channel. We use a voting threshold of 33% (1/3) for analyzing all the parietal/occipital EEG channels. More or less stringent strategies can be used by changing the voting percentage required for identifying significant time regions. Detected alpha spindle regions separated by less than 250 ms were merged together to form a single alpha spindle. Since the literature suggests that alpha spindle duration is generally between 500 ms and 2 s, isolated alpha spindle regions shorter than 250 ms were removed from the data [11]. These post-processing techniques mimic post-processing procedures used by other authors [11,12].

### SDAR model simulation study

We conducted a series of simulation studies to verify the SDAR algorithm performance in tracking model changes in time series. In these studies, an AR(2) process was simulated with varying degrees of change. In the first study, we simulated an AR(2) process where the AR coefficients changed at a known time point. The first model (Model 1) was simulated according to:

$$\begin{array}{l} x_{t}=0.6x_{t-1}-0.2x_{t-2}+\varepsilon_{t}\;\mathrm{for}\;t\in \left[1,2000\right]\\ x_{t}=0.4x_{t-1}-0.6x_{t-2}+\varepsilon_{t}\;\mathrm{for}\;t\in \left[2001,4000\right] \end{array}$$

The noise variance was set to 1 so *ε*_t_ ~ *N*(0,1). In this model, the AR coefficients change from [0.6, −0.2] to [0.4, −0.6] at time *t* = 2000. For the second study (Model 2), we simulated an AR(2) process with an increase in variance from 1 to 4 at the change point (*t* = 2000). The AR(2) coefficients were set to [0.6, −0.2] for the entire time period [0, 4000], *ε*_1,t_ ~ *N*(0,1) and *ε*_2,t_ ~ *N*(0,1):

$$\begin{array}{l} x_{t}=0.6x_{t-1}-.02x_{t-2}+\varepsilon_{1,t}\;\mathrm{for}\;t\in \left[1,2000\right]\\ x_{t}=0.6x_{t-1}-.02x_{t-2}+\varepsilon_{2,t}\;\mathrm{for}\;t\in \left[2001,4000\right]. \end{array}$$

In both simulations, the DAR model order was set to 2, and the discounting rate was set to *r* = 0.01.

### Alpha spindle simulation study using DipoleSimulator

**Figure 2:**
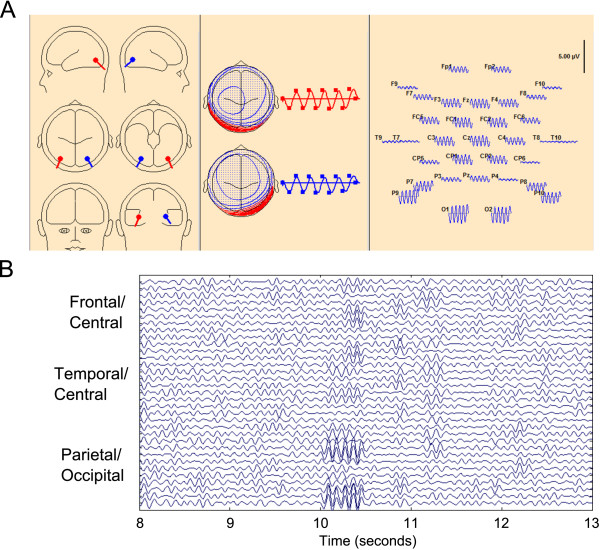
**Alpha spindle simulation results. (A)** A screen capture of the DipoleSimulator software program for simulating EEG activity. **(B)** The simulated EEG activity, with the Y-axis denoting the channel locations, ordered left to right hemisphere, frontal to occipital.

In another simulation study, we simulated EEG data using DipoleSimulator (BESA Tools version 3.3.0.4, MEGIS Software GmbH, Gräfelfing, Germany). DipoleSimulator allows the user to simulate EEG data from user-specified dipole characteristics and locations. Given the location and direction of the dipoles, DipoleSimulator simulates electrical activity that propagates through the scalp and skull. A graphical representation of our simulation is shown in Figure 2. We modeled two symmetric dipoles, located in the parietal occipital region of the brain, with the dipole direction facing the posterior of the skull (Figure 2A). The dipoles produced a 10 Hz alpha burst every 5 seconds with a duration of 500 ms, starting from the 10 s mark. Twenty bursts of alpha activity were simulated according to this model. The background RMS noise level was set to 3 *μV*. The goal of the simulation was to provide a “ground truth” dataset to compare the performance of the SDAR algorithm against.

To analyze the performance of the algorithm, we changed the signal-to-noise ratio (SNR) by changing the amplitude factor of the alpha spindle dipoles. For example, an amplitude factor of 6 means the SNR is 2 (6/3). We simulated the alpha spindles at SNR ratios of 1, 1.3, 1.6, 2 and 3 (corresponding to amplitude factors of 3, 4, 5, 6 and 9, respectively). ROC and F-measure analyses were performed for each SNR value.

The simulated EEG data was sampled at 300 Hz. The data was subsequently down-sampled to 128 Hz and band-pass filtered at [6, 15] Hz using an order 8 Butterworth filter prior to analysis. An EEG electrode mosaic with 33 channels was used for simulating the data, with a channel orientation following the international 10–10 system of electrode placement. We applied the SDAR algorithm with model order 1 and discounting rate *r* = 0.01 to the simulation output of all parietal/occipital channels, setting the voting percentage to be 33% and the fuzzy window parameter to be 0 s for comparison purposes.

### EEG data collection and processing

**Figure 3:**
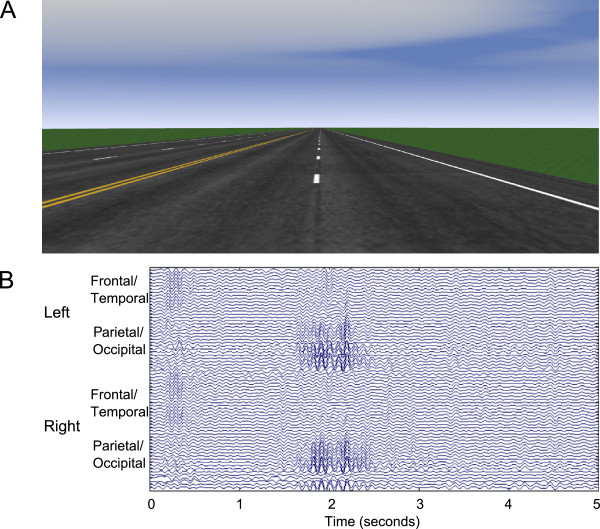
**Example data from experimental protocol. (A)** A screen capture of the simulated driving environment. **(B)** An example of an alpha spindle event in an EEG dataset, with the Y-axis denoting the channel locations in the EEG data set.

To test the efficacy of the algorithm, we applied the algorithm to two fatigue-related driving simulator datasets (Driving Data 1 and Driving Data 2) recorded from two neurologically intact, healthy, adult, right-handed and right-eye-dominant males that had been previously labeled by an expert. The two subjects had at least 10 years of driving experience prior to data collection. Informed written consent was obtained as required by the US Army Federal Regulations [36,37]. The simulation was conducted in a sound-attenuated room, and subjects were presented with a straight four-lane highway with minimal scenery (highway, roadside, and horizon) except for an occasional speed limit sign (Figure 3A). Subjects were requested to maintain the posted speed limit (25 mph or 45 mph) while keeping their vehicle in the center of the lane. A perturbation force would occasionally cause the vehicle to veer left or right in a manner similar to the effects experienced when a gust of wind crosses a real vehicle [10]. Subjects drove for approximately 70 minutes after a 10–15 minute practice period.

The EEG was recorded using a 64-channel Biosemi ActiveTwo system, and offline referenced to the average of the two mastoids. Four external channels were used to record eye movements by EOG, although EOG data was not analyzed in this study. The experiment was originally sampled at 2048 Hz and then subsequently down-sampled to 128 Hz. Figure 3B shows a sample of the resulting signal during a period of visually identifiable alpha activity.

### EEG signal preprocessing for expert identification of alpha spindles

An expert with more than 10 years of EEG processing experience visually identified and marked alpha spindle events in the EEG data. Recent literature has suggested that alpha spindling occurring in the parietal and occipital regions of the brain is related to fatigue in experiments of prolonged driving [11,38]. Therefore, the expert used EEG data from the parietal and occipital channels for identifying alpha spindle events. The expert used three different data operations to assist in the visual identification of alpha spindles: the EEG data band-passed from [1, 50] Hz, the data band-passed from [1, 15] Hz, and the Independent Component Analysis (ICA) [39-41] decomposition of [1, 50] Hz band-passed EEG data. The expert used ICA to isolate and remove eye blink and movement components if the eye activity prevented an accurate time identification of the alpha spindle events. All filters were order 8 IIR Butterworth filters from ERPLAB [42]. We used the expert’s labeled events as the ground truth to evaluate the performance of the SDAR algorithm. The expert manually marked alpha spindles in the EEG using MATLAB (The Mathworks Inc., Natick, Massachusetts) tools and functions obtained from the DETECT Toolbox [35].

### EEG signal preprocessing for the SDAR algorithm

The data was processed in EEGLAB [40] using a band-pass filter of [6, 15] Hz with an order 8 IIR filter from ERPLAB [42] before applying the SDAR algorithm. No ICA preprocessing was performed prior to this analysis. The datasets were analyzed in two passes. In the first pass, we analyzed the full data and report the detection performance and accuracy using all available information. In the second pass we partitioned the full data into two continuous equal halves for training and testing purposes. The detection performance and accuracy were reported for both the training and testing data. Results from the testing data were obtained using the optimal threshold value estimated from the training data. The receiver operating characteristic (ROC) curves and analysis of classification performance using the F-measure were performed only on the full and training data.

## Results

### SDAR model simulation results

**Figure 4:**
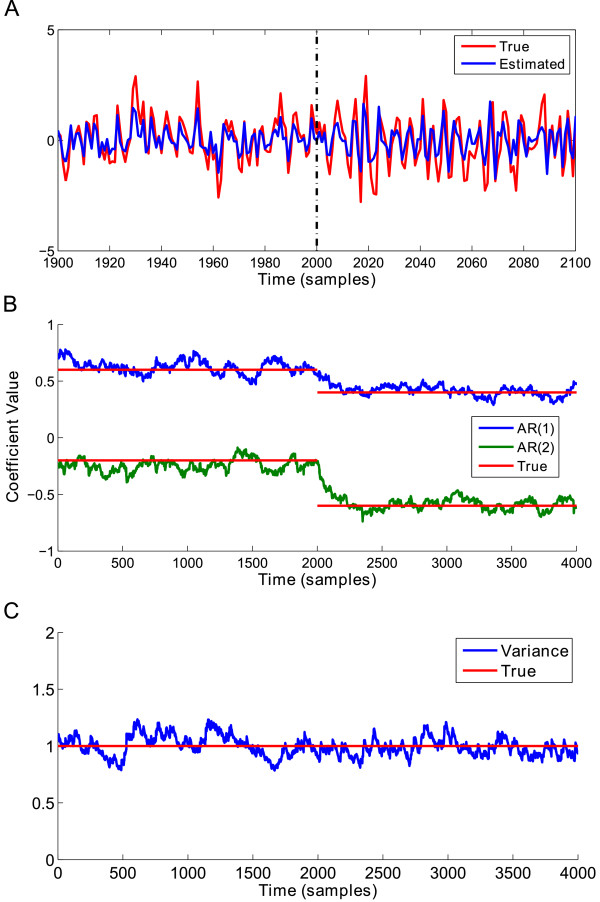
**AR model 1 simulation results. (A)** A plot of the simulated AR(2) signal for the time range [1900, 2100] for readability. Blue = original signal, Red = estimated signal, Dashed vertical line: time of the change point at *t* = 2000. **(B)** The plot of the estimated AR(2) coefficients over-plotted with the known true value of the AR(2) coefficients. **(C)** The plot of the estimated variance *σ*^2^ over-plotted with the known true value of the variance.

Our first analysis verifies SDAR algorithm performance in tracking changes in underlying AR model parameters. A plot of the results of the simulation for Model 1 is shown in Figure 4. Figure 4A shows a plot of the true signal in red, with the estimated signal from the SDAR algorithm in blue. We plot the time region around the change point [1900, 2100], as well as plotting the change point time location (dashed black line) for readability. Here we see that the SDAR algorithm can accurately capture the change in dynamics of the time series. The estimated AR(2) coefficients accurately track the true values within 100 samples of the change point *t* = 2000 (Figure 4B). The variance of the model remained constant in this simulation, and so the estimated variance should be close to the true value for the entire time period (Figure 4C).

**Figure 5:**
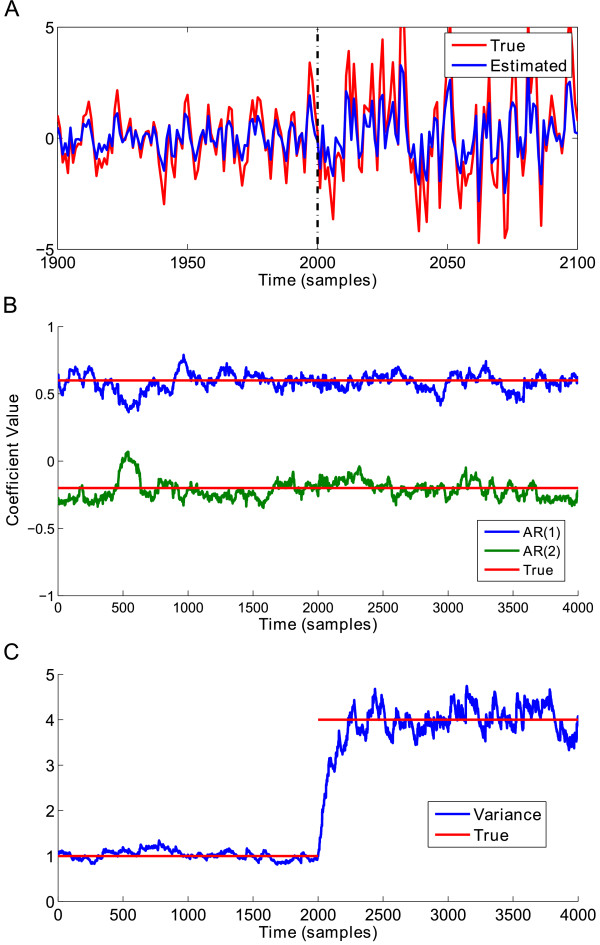
**AR model 2 simulation results. (A)** A plot of the simulated AR(2) signal for the time range [1900, 2100] for readability. Blue = original signal, Red = estimated signal, Dashed vertical line: time of the change point at *t* = 2000. **(B)** The plot of the estimated AR(2) coefficients over-plotted with the known true value of the AR(2) coefficients. **(C)** The plot of the estimated variance *σ*^2^ over-plotted with the known true value of the variance.

The results for the simulation of Model 2 are shown in Figure 5. As before, the SDAR algorithm was able to accurately approximate the true time series signal (Figure 5A and B) and correctly detected a change in the signal variance at the change point *t* = 2000 (Figure 5C).

### Simulated alpha spindle detection performance

**Figure 6:**
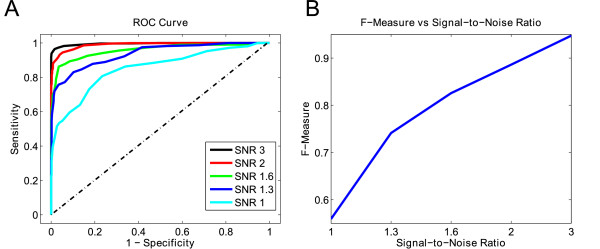
**Alpha spindle detection performance on simulated data. (A)** Plot of the ROC curves for each of five different SNR values at *β* = 2. **(B)** Plot of the F-measure versus the SNR.

Figure 6 displays the performance of the SDAR algorithm in detecting alpha spindles in the simulation study using DipoleSimulator. Figure 6A shows the ROC curves for each of the five simulated SNR datasets, while Figure 6B shows the F-measures at each SNR value. The performance of the algorithm increases with increasing SNR values, achieving an F-measure value of approximately 0.95 at SNR of 3. These results suggest that spindle activity at SNR values of 2 or greater can be reliably detected using the SDAR algorithm.

### Alpha spindle detection in real EEG data

We then analyzed the two datasets obtained from the simulated driving experiment (see Methods). These datasets are referred to as Driving Data 1 and Driving Data 2, respectively. We use an order 1 SDAR model with discounting rate *r* = 0.01 and 33% (1/3) voting percentage for the duration of the paper. The results of using an order 2 SDAR model were similar, and are included in the Additional file 1 included with this manuscript. Our first analysis compares the expert labeled data to the SDAR algorithm’s labeled data using a fuzzy window parameter of 0 s. Detected spindles of less than 250 ms were removed prior to analysis, while spindles separated by less than 250 ms were merged to form one spindle. An online implementation of this algorithm would have a delay of at least 250 ms (to verify that the activity is a spindle), plus any additional computational, data acquisition, and processing overhead. The processing of each channel can be done in parallel, reducing the computational burden.

Results of the analysis are shown in Table 1. In the Full Data condition, the SDAR algorithm detected 138 of 141 total alpha spindles, for a hit rate of 97.87%. The Spindle Temporal Error (STE), which is the total time in the False Negative condition divided by the total number of alpha spindle events, summarizes the temporal localization accuracy of the algorithm. For the Full Data analysis, the STE is approximately 100 ms, indicating excellent temporal resolution of the alpha spindle detections. Analysis of the Testing Data (using the training data parameters) showed similar results. This section only contained 45 alpha spindles, of which 42 were correctly identified. The STE for this condition is approximately 150 ms. The sensitivity, specificity, and precision values are roughly constant in the two halves (Training and Testing) parts of the datasets. We also report the total time in each of the 4 states (Agreement, Null Agreement, False Positive, False Negative) as reported by the compareLabels function from the DETECT Toolbox.

**Table 1:** Classification performance of the algorithm versus the ground truth events for Driving Data 1 for three data conditions: the full data without partitioning, the training data and the testing data, respectively

	**Full data**	**Training data**	**Testing data**
*Sensitivity/Recall*	.915	.895	.863
*Specificity*	.966	.966	.984
*Precision*	.536	.612	.581
*Hit Rate*	97.87% (138/141)	100% (96/96)	93.33% (42/45)
*Spindle Temporal Error*	~96 ms	~120 ms	~150 ms
*Agreement*	146.430 s	98.617 s	43.055 s
*Null Agreement*	3591.156 s	1762.414 s	1861.141 s
*False Negative*	13.586 s	11.531 s	6.813 s
*False Positive*	126.828 s	62.445 s	31.000 s

A fuzzy window parameter of 0 s was used.

Note that a fuzzy window parameter of 0 s indicates that very minor differences in labeled regions will count negatively against the performance of the algorithm. It is unrealistic in practice to assume an exact temporal agreement between an expert and the algorithm, or even among two different experts. Incorporating an allowable timing error in the comparison can produce a more appropriate comparison.

**Figure 7:**
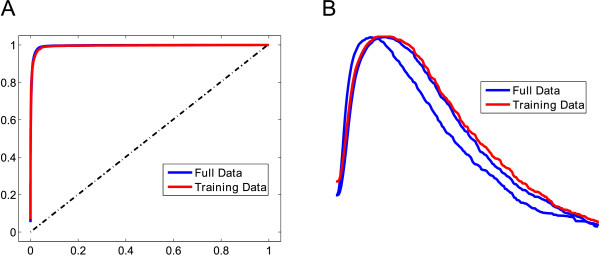
**Alpha spindle detection performance on real data. (A)** Plot of the ROC curve for the alpha spindle detection algorithm for the Full Data and Training Data of Driving Data 1 using a fuzzy window parameter of 100 ms. **(B)** The corresponding modified F-measure plot for the ROC curves shown in **(A)**.

When using a fuzzy window parameter of 100 ms (meaning errors less than 100 ms before or after the events are treated as agreements), the performance of the algorithm significantly increases (Table 2, Data 1 columns). While the hit rate remained unchanged in the Full Data (137/141), the False Negative time significantly decreased to only 7.438 s of the data (from 13.586 s), resulting in an improved STE of about 52 ms. This indicates that many False Negative errors are associated with small timing differences between the expert and the algorithm; these small differences may be negligible in real EEG analysis. A majority of the timing error is accounted for by using the fuzzy window parameter. The ROC curve and F-measure plots are shown in Figure 7. The analysis for Driving Data 2 is also shown in Table 2.

**Table 2:** Classification performance of the algorithm versus the ground truth events for Driving Data 1 and Driving Data 2 for three data conditions: the full data without partitioning, the training data and the testing data, respectively

	**Full data**		**Training data**		**Testing data**	
	***Data 1***	***Data 2***	***Data 1***	***Data 2***	***Data 1***	***Data 2***
*Sensitivity/Recall*	.957	.876	.959	.914	.952	.904
*Specificity*	.981	.964	.974	.958	.989	.934
*Precision*	.704	.620	.706	.660	.701	.400
*Hit Rate*	97.16% (137/141)	94.36% (184/195)	100% (96/96)	95.38% (124/130)	91.11% (41/45)	93.85% (61/65)
*Spindle Temporal Error*	~52 ms	~114 ms	~50 ms	~77 ms	~40 ms	~96 ms
*Agreement*	165.422 s	157.008 s	114.539 s	106.688 s	50.833 s	59.047 s
*Null Agreement*	3635.516 s	2584.453 s	1167.875 s	1257.414 s	1866.859 s	1275.273 s
*False Negative*	7.438 s	22.195 s	4.875 s	9.984 s	2.563 s	6.273 s
*False Positive*	69.625 s	96.344 s	47.720 s	54.922 s	21.703 s	89.414 s

A fuzzy window parameter of 0.1 s was used.

**Figure 8:**
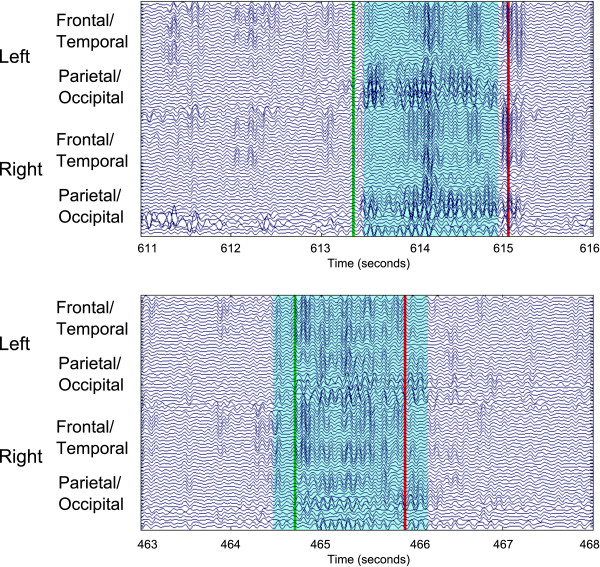
**A plot comparing expert-labeled alpha spindles with alpha spindles labeled by the SDAR algorithm.** The algorithmic labelings are shown in light blue, while the start and end event codes (in green and red, respectively) denote the expert-labeled regions. (Top). An example where the algorithm labels a slightly narrower region as alpha spindle when compared to the expert. This minor timing difference will generate False Negative errors if no fuzzy window parameter is used. (Bottom). An example where the algorithm over-estimates the alpha-spindle region. This will generate False Positive errors, which can be accounted for if a fuzzy window parameter is used.

An example detection in the testing data of Driving Data 1 is shown in Figure 8. The shaded cyan region denotes the labeled region by the algorithm, while the start and end event codes denote the expert labeled alpha spindle regions. Figure 8 (Top) shows an example where the algorithm labels a slightly narrower region when compared to the expert. A fuzzy window parameter of 0 s will generate two False Negative decisions (for both the start and end time of the alpha spindle being incorrect) for a total of about 150 ms. A similar situation is shown in Figure 8 (Bottom), where the algorithm over-estimates the alpha spindle region. This will generate two False Positive decisions with the total error approximately 200 ms. In both cases, the algorithm and the expert agreed that an alpha spindle was present; only minor timing errors in the start and end times of the alpha spindle were present. A fuzzy window parameter of 100 ms (meaning 100 ms before and after agreement regions are treated as agreements) eliminates most of the error in these detections and provides a more appropriate summary of the performance.

### Comparison with other alpha spindle detection measures

**Figure 9:**
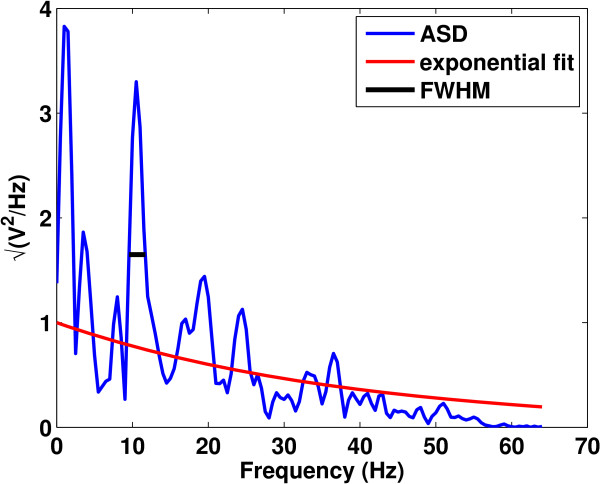
**An illustration of the technique used in **[11]**for detecting alpha spindles in EEG.** The blue curve is the Amplitude Spectral Density (ASD), the red is the exponential fit to the data, and the black line is the full-width at half maximum (FWHM) of the peak amplitude.

Previously, Simon et al. [11] introduced a technique for detecting alpha spindles in EEG based on calculating the amplitude spectral density (ASD) of a Hamming-windowed EEG signal in a sliding window (using 1 s windows with a 250 ms temporal slide). The ASD algorithm then finds the maximum peak frequency in [3, 40] Hz in each window. If this peak is in the alpha range [8, 13] Hz, the ASD algorithm calculates the full-width at half maximum (FWHM), which is the width of the peak at half the maximum amplitude. If the FWHM is less than twice the noise bandwidth (NBW) of the Hamming window (2 × 1.37), the ASD algorithm identifies the time region as an alpha spindle. The algorithm also calculates the oscillation index, which is the ratio of the area under the peak in the FWHM range and the area under an exponential fit of the data in the same FWHM range as a measure of the signal-to-noise (SNR) of the alpha spindle. The exponential fit is used to estimate the 1/*f*-like noise found in EEG [43]. In our implementation we fit an exponential of the form:

$$f\left(x\right)=\exp\left(\frac{x}{\gamma }\right)$$

where *γ* is the shape parameter of the exponential function. We estimate *γ* by minimizing the sum of squared errors:

$$\hat{\gamma }={\mathrm{argmin}}_{\gamma }\left(\overset{N}{\underset{i=1}{\sum }}{\left(y_{i}-f\left(x_{i};\gamma \right)\right)}^{2}\right)$$

where *x*_*i*_,*y*_*i*_ is the pair of fitted and observed values of the ASD, respectively. An example for a time segment with an alpha spindle is shown in Figure 9. Here, the peak frequency is located at approximately 11 Hz. The FWHM of this spectrum is less than 2.74, and so the segment is treated as an alpha spindle.

We compare the ASD and SDAR approaches for Driving Data 1. In order to compare SDAR with ASD, we applied the ASD algorithm as follows. For the ASD algorithm we processed the data according to the procedure used in [11] (128 Hz sampling rate and [0.5, 48] Hz band-pass filter, ICA to minimize muscle and eye artifacts). For comparison purposes with the SDAR algorithm, we also applied the ASD algorithm without ICA artifact removal. For the SDAR algorithm we processed the data only using a [6, 15] Hz band-pass filter. We chose only one channel, PO7, in our analysis as this produced the highest detection accuracy for single-channel detection with the ASD algorithm. We used a fuzzy window of 0.1 s.

The results of the comparison are shown in Table 3. The ASD algorithm without ICA artifact removal has sensitivity, specificity and precision values that are significantly lower than those of SDAR. The ASD algorithm detects 78% of the alpha spindles in the data with an STE of 478 ms. After ICA artifact removal, the performance of the ASD algorithm improved in specificity and precision with a slight reduction to sensitivity. ASD with ICA artifact removal achieved a slightly lower hit rate (67%) when compared to without ICA. In comparison, SDAR modeling has higher sensitivity/specificity/precision values as well as a higher overall hit rate.

**Table 3:** Comparison between the SDAR algorithm fitting and the ASD algorithm for channel PO7 of Driving Data 1

	**SDAR**	**ASD algorithm without ICA**	**ASD algorithm with ICA**
*Sensitivity/Recall*	.942	.607	.529
*Specificity*	.984	.728	.909
*Precision*	.728	.094	.209
*Hit Rate*	97.16% (137/141)	78.72% (111/141)	66.67% (94/141)
*Spindle Temporal Error*	~150 ms	~478 ms	~560 ms
*Agreement*	157.008 s	104.063 s	88.961 s
*Null Agreement*	3584.453 s	2700.063 s	3373.461 s
*False Negative*	22.195 s	67.320 s	79.359 s
*False Positive*	96.344 s	1006.555 s	336.359 s

A fuzzy window parameter of 0.1 s was used.

## Discussion

In this paper we propose an efficient method for detecting large narrowband increases in oscillatory EEG activity using change point detection methods based on discounted autoregressive models. This technique was applied to the alpha frequency range where the goal of the method was to detect alpha spindling activity and to estimate features of the alpha spindling such as the spindle rate and temporal localization. Our results show that this approach successfully identifies alpha spindles in EEG time series with good time resolution, allowing for the possibility of using characteristics such as alpha spindle frequency and duration as features for other types of modeling approaches, including state classification, fatigue monitoring, and performance prediction.

Early work using change point detection models for EEG data analysis was done by Brodsky et al. [44]. These authors use nonparametric modeling techniques for the analysis of alpha activity with eyes closed versus eyes open. In contrast to their work, our work uses parametric modeling of the EEG using the DAR model to detect oscillatory activity. As the EEG is naturally time-dependent, methods based on autoregressive models are appropriate representations of the dynamics of EEG. Our use of the DAR model is especially attractive in that the model can adapt to non-stationary time series, a feature that is often present in EEG. Our approach is also computationally efficient, only requiring a few matrix operations at each iteration of the algorithm, making it an attractive analysis technique for large EEG datasets.

There are some differences in processing between our SDAR method and the ASD method proposed by Simon et al. [11] that deserve mention. First, our method band-passes the data to [6, 15] Hz, while the ASD method uses a [0.5, 48] Hz band-pass. The band-pass from [6, 15] Hz effectively removes muscle in the higher frequency bands, while limiting the impact of eye-related artifacts in the lower frequency bands. This reduces the need for extensive artifact removal post-processing with ICA [41] and auto-regressive techniques [45] as used in [11]. Also, the effects of eye-related movements in EEG are minimal, since we are only analyzing parietal and occipital channels. Finally, our approach is not based on frequency characteristics of the signal other than the initial [6, 15] Hz band-pass. Because of this, we are not limited by the time-resolution of FFT-based methods in short time windows.

The SDAR approach sequentially calculates a quadratic loss score at each time point and uses this score function to identify irregular periods in the data. We obtain an effective time resolution equal to the window size of our temporal smoothing function. One possible disadvantage of our approach is that it requires *a priori* knowledge of the frequency range of interest before analysis. This may not be much of a concern for the analysis of EEG, since researchers often use well-understood, predetermined bands for analysis.

One important parameter of the SDAR model is the discounting rate, *r*. This parameter controls the level of contribution past data points have in the current model. If the discounting rate is large (> .05) the model adapts very quickly to the new dynamics of the data, making detecting unusual behavior difficult. It also is less robust to minor variations in the signals which are not statistically significant. Therefore we suggest using slower learning rates to prevent the model from learning too much of the alpha spindle behavior while still adapting to slowly changing behavior. We found that discounting rates between 0.01 and 0.001 perform well in isolating alpha spindles of data sampled at 128 Hz. These learning rates would need to be adjusted for different sampling frequencies: the rates should be decreased when the sampling rate increases and the rates should be increased when the sampling rate decreases.

Previous research in autoregressive modeling of EEG data has shown that large model orders are needed to estimate the underlying dynamics of the EEG signal. For example, [46] used AR models of order 10 to analyze the spectral contents in short EEG signals, while [47] used AR models of order 6 for distinguishing among several different mental tasks. In contrast, our work has shown that using low model orders are sufficient to identify statistically irregular EEG data segments. A possible explanation for this is the fact that we narrowly band-pass the EEG signal to the alpha frequency range prior to analysis. This band-pass reduces the frequency content of the signal. Because of this, only a few model orders may be needed to capture the oscillatory dynamics of this narrow band signal and discounting captures model variation. For EEG data filtered at a wider band, more orders are likely needed. Another possible explanation is that we are primarily focused on *when* EEG data is statistically irregular and not necessarily *why* it is statistically irregular. A low order representation of the EEG may be sufficient purely for identifying when sections of data are statistically irregular.

Several techniques have been proposed for detecting oscillatory activity in higher frequency bands. For example, a technique based on FFT analysis in the [80, 500] Hz frequency range was proposed in [48]. Another study of higher frequency data [49] proposed a technique for oscillatory event detection based on amplitude and duration thresholding of a short-time line length function. This technique is similar to our approach in that the short-time line length function bears a large degree of similarity to the order 1 autoregressive structure used in this work. Another technique based on an adaptive Hilbert Transform has been proposed for oscillatory EEG analysis in neonates [50]. An adaptive Hilbert Transform could be used to capture a time-dependent amplitude envelope which could be thresholded to find alpha spindles. In contrast to these previous works, our work is based on an adaptive statistical representation of the alpha-band EEG.

### Future applications

The SDAR algorithm is based on an adaptive statistical representation of the EEG time series and is not limited to alpha spindle detection in EEG. Results of our simulation study show that alpha spindles can be detected reasonably well if the spindle signal strength is at least 50% stronger than then the background noise signal (Figure 6). This suggests that this algorithm can be potentially used to detect oscillatory activity in other EEG frequency bands if the expected SNR of the activity is at least 1.5. For example, Craig et al. [51] analyzed EEG oscillatory activity in a simulated driving paradigm and showed that as the subject tired, oscillatory activity in both theta and alpha bands increased over the entire cortex, while activity in the delta band showed no significant changes. Fast wave activity also showed a significant increase primarily in frontal areas.

Other types of oscillatory activity could be modeled using our SDAR approach. For example, oscillatory activity in the theta range was analyzed by Cruikshank et al. [52] using wavelet-based techniques to analyze cortical activity underlying sensorimotor integration in humans. High frequency oscillatory activity is also a major area of interest in epilepsy research [48]. As new experiments are conducted at increasing sampling rates, oscillatory activity in several different EEG bands can be analyzed to find spatiotemporal patterns across a range of experimental paradigms [53]. Our technique based on SDAR modeling of the EEG can be applied to find spatiotemporal patterns of changes that may be indicative of mental state changes. This will be investigated in future research.

Our method requires an initial band-pass of the EEG data in a relevant frequency range of interest prior to analysis. For our analysis we band-passed the data at [6, 15] Hz for detecting alpha spindle oscillations in EEG. Since eye movement artifacts in EEG are generally in frequency ranges smaller than 6 Hz and artifacts from muscle movements are generally greater than 15 Hz, no additional artifact preprocessing is required. However, analysis of other brain regions may require additional preprocessing to remove artifacts prior to analysis. This is especially true for [3, 7] Hz theta oscillations, as eye movement artifacts will be more prevalent and pervasive when compared to the analysis of the alpha band. Analysis of the gamma frequency range will require removal of high frequency muscle activity as well as the removal of power-line noise (either at 50 or 60 Hz) prior to modeling by the SDAR algorithm.

Our primary goal of this work was the development of an algorithm for accurately identifying alpha spindles in EEG. We focused primarily on the parietal-occipital EEG channels as alpha spindles generally occur in these regions. However, this approach could be applied to analyze all the EEG channels simultaneously. In this way, the changes in the EEG could be correlated across brain regions, thus revealing additional features that can be useful for analysis. This is currently the topic of future research.

Recent advances in sensor technologies have enabled the non-invasive recording of neural activity in a variety of scenarios [38,54] with some technologies aimed at improving performance in healthy individuals [55]. Efficient methods for detecting and identifying changes in EEG oscillatory activity may have practical applications in BCI contexts. For example, our work in detecting alpha rhythm changes (alpha bursts/spindles) may facilitate early detection of fatigue onset before lapses or microsleeps occur [7,9-12,38] and may be useful for preventing potentially dangerous situations such as attention lapses or microsleeps during tasks that require sustained levels of vigilance. Parameters that may be used for analysis of rhythmic alpha activity include frequency range, duration, rate, topography and peak frequency [11,12,56]. Another area where our approach might be useful is for real-time online monitoring of sleep. Rechtschaffen and Kales [57] published guidelines for manually scoring stages of sleep, including criteria for scoring alpha spindles in the form of k-complexes observed during stages I-II of sleep. Automated methods for sleep stage scoring based on the R&K gold standard visual scoring of EEG recordings have been published; however, none of these methods have reached the level of acceptance to the extent of R&K [56]. An automated system that is robust and validated is still lacking today.

## Conclusion

In this work we showed that discounted autoregressive models can be used to model the alpha band EEG time series for detecting alpha spindle events in EEG. Our method is based on statistical principles and can generally be applied to detect rhythmic activity in any frequency band or brain region. As the algorithm is based on a time-adaptive statistical representation of the signal, it can account for slowly non-stationary behavior, making it an attractive model for EEG data analysis.

## Authors’ contributions

Designed the software used in the analysis: VL, KAR. Conceived and designed the experiments: SK. Performed the experiments: SK. Analyzed the data: VL, SK, KAR. Wrote the paper: VL, SK, KAR. All authors read and approved the final manuscript.

## Supplementary Material

Additional file 1 Analysis of SDAR Model Order Performance. Click here for file
