# Supplementary material for: Detecting alpha spindle events in EEG time series using adaptive autoregressive models
Source: BMC Neurosci. 2013 Sep 18;14:101. doi: 10.1186/1471-2202-14-101 (PMC3848457; doi:10.1186/1471-2202-14-101)
Supplement: Additional file 1 — Analysis of SDAR Model Order Performance. [file 1471-2202-14-101-S1.docx]

Additional file 1:

**Analysis of SDAR Model Order Performance**

An order 1 SDAR model can track unusual changes in the amplitude of a signal. Alpha spindles generally create large fluctuations in amplitude and can therefore be identified by detecting statistically significant variations in the amplitude. However, alpha spindles also may create a large fluctuation in frequency, which should also be detectable by using an order 2 model. We have re-analyzed our data using an order 2 model for Driving Data 1 and show that the performance of the algorithm is similar to that of the order 1 model, with a few exceptions.

Supplementary Table 1. Comparison between the SDAR algorithm for different model orders for channel PO7 of Driving Data 1. A fuzzy window parameter of 0.1s was used.

|  | SDAR  Order 1 | SDAR  Order 2 |
| --- | --- | --- |
| *Sensitivity/Recall* | .942 | .923 |
| *Specificity* | .984 | .938 |
| *Precision* | .728 | .418 |
| *Hit Rate* | 97.16% (137/141) | 99.29% (140/141) |
| *Spindle Temporal Error* | ~150ms | ~97ms |
| *Agreement* | 157.008s | 165.188s |
| *Null Agreement* | 3584.453s | 3469.117s |
| *False Negative* | 22.195s | 13.719s |
| *False Positive* | 96.344s | 229.977s |

In sensitivity and specificity both model orders 1 and 2 perform about the same. While the order 2 SDAR model was more accurate (higher hit rate, higher agreement time, lower False Negative time), the order 2 SDAR model also had a significantly higher False Positive time (from 96 seconds to about 230 seconds). This makes intuitive sense for several reasons. First, alpha spindles generate large fluctuations in both amplitude and frequency; using an order 2 model (which now enables tracking of statistically significant amplitude and frequency changes) improves the overall accuracy of the algorithm. However, there may be background non-spindle alpha frequency changes that are detected by the algorithm (resulting in a higher false positive rate). Which model order is best will depend on the context of the analysis performed.
